# Supplementary material for: English colour terms carry gender and valence biases: A corpus study using word embeddings
Source: PLoS One. 2021 Jun 1;16(6):e0251559. doi: 10.1371/journal.pone.0251559 (PMC8168888; doi:10.1371/journal.pone.0251559)
Supplement: Attachment — Submitted filename: PLOS_ONE_gender.pdf [file pone.0251559.s001.pdf]

Review of "Colour terms carry gender and valence biases in natural language corpora"

The reviewer identifies himself as Bodo Winter and is available for follow-up questions.

This is a fun paper that makes a useful contribution. I definitely think that this is definitely PLOS ONE material, as it uses a rather innovative methodology and as the paper covers a topic that is of interest to an interdisciplinary readership. There are a few shortcomings in how the methods are presented, as well as perhaps some gaps with respect to the theoretical framing — but nothing that couldn't be changed relatively easily. I would hope that this paper gets accepted once the changes have been implemented.

Presentation of methods and results

While the online form mentions the word embeddings used, I could not find any links to the analysis code and data used for this paper, including code that reproduces the plots. The analyses are thus not strictly speaking reproducible, and there should be a data availability statement at a prominent position in the methods section that links to the code that produces all numbers and plots that lie behind the results.

I am somewhat familiar with NLP, word2vec etc., but I found it still hard to follow the way the results are presented. For one, the figures of the histograms in the supplementary materials should perhaps be part of the body of the paper. They are quite integral to understanding what's going on, especially since much of the methods section talks about using 2SD and 3SD cut-off values from the distribution — without the actual distribution ever being shown in the paper!

Overall, I think that a lot of the presented numbers need to be "grounded", that is, the reader wants to get a sense for the numbers involved, and what are large or small quantities. Given that the main topics of the paper (gender, color, valence) are of high relevance to psychologists, this audience will definitely want to see some measure of effect size that can readily be interpreted. Throughout the paper I was left wondering whether these were strong or weak biases.

The inferential statistics are based on resampling (as far as I understood), which is quite cool, and it seems to make sense for this dataset. But even though I've used resampling techniques myself quite a bit, I felt that the logic of the resampling techniques on p. 8-9 could be explained in more detail, especially since many psychology-oriented readers will not be familiar with this.

I think it would also be helpful to present tables of the gender and valence bias values. While the ranking of the color words is inferable from Fig. 1, I think people would like to see the actual values and the ranking in a simple table.

Now, I like the approach that is presented in the paper, and it makes a lot of sense for me to do this for gender, since there are no available gender norms for color words. However, there *are* valence norms for color words, such as the widely used Warriner et al. (2013) dataset. I recognize that these are very different quantities from the ones presented in the

text, as this is not text-inferred norms but humanly rated valence. That said, I think it's important to consider this data, since it is there and readily available. I did a simple check and found that in fact all color words were biased towards positivity in this dataset. Pink, orange, and blue were among the most positive, and black is negative. A lot of this (although not all) mirrors the results obtained in this paper. I think correlating the values from the paper with this widely used dataset would be useful, if only for triangulating the results. Any differences that emerge could be theoretically interesting.

```
col_val
# A tibble: 10 x 4
   Word      Val Val_z Percentile
   <chr>   <dbl> <dbl>      <dbl>
 1 black   5.4   0.26       0.580
 2 blue    6.53  1.15       0.88
 3 brown   5.52  0.36       0.62
 4 green   6.29  0.96       0.83
 5 orange  6.81  1.37       0.93
 6 pink    6.68  1.27       0.91
 7 purple  5.6   0.42       0.65
 8 red     5.67  0.48       0.67
 9 white   6.18  0.88       0.81
10 yellow  6.09  0.8        0.79
```

I attached the code and dataset used to reproduce this table in this zip file:

http://appliedstatisticsforlinguists.org/color_valence_papers_code.zip

There's also some NLP work on word-color associations by Muhammad Saif that I think is worth incorporating, and he has a whole database of words that are associated with color terms. Perhaps one could do the reverse here and look for the gendered terms in this database and see whether they are associated with the colors.

https://www.aclweb.org/anthology/W11-0611.pdf

I'm not saying that the latter work has to be incorporated, but it's worth acknowledging it. Incorporating the Warriner et al. (2013) data however is going to be a piece of cake, so perhaps worth doing.

Theoretical framework

The paper cites a good deal of relevant research. I think it is missing out however on a bit of a stronger connection to cognitive science. Specifically, there are theoretical approaches within cognitive science that *predict* that biases found in psychological studies should be reflected in linguistic data, and work in cognitive science which looks at how language statistics in general reflect psychological biases and real-world patterns. Chiefly, Max Louwerse's Symbol Interdependency Theory comes to mind. We review some of the relevant literature in section (iii) "Language statistics as knowledge" of this paper (pp. 4-5): https://royalsocietypublishing.org/doi/pdf/10.1098/rstb.2017.0137

I'm not saying that you have to cite our paper, but perhaps you find some of the references therein useful. I would think that by incorporating insights from this general view of language (as statistically mirroring psychological biases) into your work, you could show scope and increase the relevance of this paper to a wider audience.

There's also this paper that I think will turn out relevant to you:

Shepard, R. N., & Cooper, L. A. (1992). Representation of colors in the blind, color-blind, and normally sighted. Psychological Science, 3(2), 97-104.

It shows that semantic associations between colors are similar in the blind, even if they have never seen colors. This can only be explained by making recourse to language statistics.

Finally, I have attached two papers from our lab into the zip file above (http://appliedstatisticsforlinguists.org/color_valence_papers_code.zip), in one we look at gendered terms in natural language corpora and show that the frequency with which terms are mentioned as male/female significantly correlates with psychological biases from a gender bias rating study. The paper is at proof stage, but it is citeable — I think that this result is directly relevant to what you are doing.

When it comes to other cognitive science work, the finding that white > black in terms of positivity is interesting to me, and there's much work this relates to. In the zip file, I attached a paper of mine that reviews the psychological work on black/white darkness related metaphors. And when you state that "black" is associated with sadness and death, it is critically also associated with fear (as evidenced by many studies).

Sorry for recommending that many of my own papers — there's no need to cite them (the paper is good as is), but the references therein will surely prove useful for you to consider enlarging the scope of your work and incorporating some of the cognitive science work into your introduction and discussion. Your paper is quite on the short side of things anyway!

Other concerns

I agree that for large-scale corpora such as Wikipedia and newspapers, author and register variation etc. does not matter that much (as discussed in your limitations section). However, when it comes to the news in particular, it is quite likely that some of the metaphorical meanings of these words are quite dominant. For example, I can imagine that "black" in particular comes up in quite heated racial discussions, and some of its negativity associated with this word is either due to structural biases against blacks (e.g., reporting of crimes etc.) or newspaper articles discussing the negative topic of racism. Likewise, "green" in newspaper texts will almost always be associated with environmentalism. It's perhaps worth noting more strongly that newspapers are by no means a 'neutral' sort of text type. You mention this in your limitations section a bit, but I think this could be emphasized more strongly, perhaps also with specific examples.

Speaking of examples: a linguist reader would perhaps want to see SOME linguistic examples from the corpora to see how the gender/valence dynamics of these words pan out in actual discourse. This would make things MUCH more concrete for some readers.

The paper is generally pretty good in terms of political correctness (important for this topic!), but I feel that there are two minor areas for improvement:

- Include a statement somewhere that you recognize that gender is a non-binary concept. I'm all for using the categories 'masculine' and 'feminine' as this is arguably the primary division within conceptual gender space, but people from the LGTBQ+ community may find it offensive if the non-binariness of gender is not at least acknowledged (in the paper attached in the zip, we do this at length in footnote 1).

- Some statements have to be hedged more to make it clear that you do not endorse them. This starts with the opening sentence "Many people would agree that *blue* is for boys and *pink is for girls*" — sounds like we should agree with this! Reframing things in terms of "stereotypes" is, I think, better: "In Western societies, blue is stereotypically associated with boys and pink is stereotypically associated with girls". Likewise, "many parents are used to choosing pink when dressing their daughters" could be misread to sound as if this is a good habit. Just saying "many parents chose pink…" is better. Finally, "Pink further represents groups of low social power, such as children and homosexuals", I would change to "… is stereotypically associated with groups ...". Also, consider using another term for 'homosexuals', which some consider to be quite negative (it is not the term the community uses to refer to itself).

Minor points

- Supplementary materials: "that blue is 0.08 more positive than red" — but what is the metric here? Is this small or large?
- P. 3, "Empirical studies have demonstrated" — empirical studies with whom? This is super relevant here since this bias is presumably not universal and the study populations matter here.
- P. 3, "because gender (girl) repeatedly co-occurs with the actual color (*pink*)" -> The italics are used for words elsewhere, but if this is the actual color, why is this italicized? Also, "gender" (which is an abstract concept) cannot "co-occur" with a color… this is a bit of a category error.
- P. 4, I'm not all that happy about capitalizing artificial intelligence and natural language processing. Also, do we really need to talk about the approach in terms of AI and isn't this a bit buzz-wordy? At this stage, I think we can just identify the precise approach right away. NLP and AI are super common after all.
- P. 4, Should "nurse" and "housekeeper" be in italics?
- P. 5, "pushing its valence bias towards neutrality" -> Wait, if it occurs in highly positive and highly negative contexts, it's not neutral, right? Do you perhaps mean the average?
- P. 5, "the literature on gender stereotype***s***" — s missing

- P. 5 — you mention first “typically 300” then the precise number of dimensions. The first is vague (I immediately asked myself: so how many dimensions were used here?), and it’s a bit confusing to have them both so close to each other.
- P. 6, “We can test words using the following cosine similarity function” — How is this a “test”? That’s a bit of a weird word here?
- P. 7, Can you avoid using the word “antithetical” which many non-native speakers won’t know?
- P. 7, “of the two dimensions ***(gender and valence)***” — clearer to add this right away
- P. 8 — this page and the section “Data Analysis” needs the most reworking. First, I took issue with “Therefore, we were able to test if any of our words of interest were statistical outliers”, which does not follow from the distributions being normal. One can test for outliers for any distribution!! It’s not that obtaining a particular distribution is intrinsically connected to outlier-detection. Also, it has to be flagged that 2SD and 3SD are “heuristic cut-offs” -> These are really arbitrary standard deviation values that we have historically inherited. You are using this as a heuristic criterion, and that’s ok, but I’d like the word “heuristic” to be used to make it clear that this is a definitional matter, depending on where one sets the boundary. At first when you said “the 100,000 most common words” it wasn’t clear to me where these come from. And when you reference “the population mean”, I’m not sure you can say this unless you really have the full population (which you don’t, since even a corpus is a sample).
- P. 12, “numerically, but not statistically” — drop the “but not statistically” bit, since this seems to equate inferential statistics with statistics… which I’m not OK with ;-)
- P. 12, “while the findings on *blue* went against the notion that *blue* signals masculinity” — not sure… first, “signals” may not be the right word here, since what is in question is just whether language statistics reflect this bias. Second, absence of evidence is not evidence of absence, and so I wouldn’t say that these results go *against* this notion.

Good stuff! Looking forward to seeing this paper revised and published!
