# Supplementary material for: English colour terms carry gender and valence biases: A corpus study using word embeddings
Source: PLoS One. 2021 Jun 1;16(6):e0251559. doi: 10.1371/journal.pone.0251559 (PMC8168888; doi:10.1371/journal.pone.0251559)
Supplement: Attachment — Submitted filename: Response_letter_PLoS.docx [file pone.0251559.s002.docx]

**Reviewer: 1**

R.1.1. The reviewer identifies himself as Bodo Winter and is available for follow-up questions.

This is a fun paper that makes a useful contribution. I definitely think that this is definitely PLOS ONE material, as it uses a rather innovative methodology and as the paper covers a topic that is of interest to an interdisciplinary readership. There are a few shortcomings in how the methods are presented, as well as perhaps some gaps with respect to the theoretical framing — but nothing that couldn’t be changed relatively easily. I would hope that this paper gets accepted once the changes have been implemented.

**Reply: We thank Dr Winter that he shares our enthusiasm for this kind of research and our current study in particular. We appreciate his insightful suggestions. Below, we address the specific comments regarding method presentation and theoretical framing.**

R.1.2. Presentation of methods and results

While the online form mentions the word embeddings used, I could not find any links to the analysis code and data used for this paper, including code that reproduces the plots. The analyses are thus not strictly speaking reproducible, and there should be a data availability statement at a prominent position in the methods section that links to the code that produces all numbers and plots that lie behind the results.

**Reply: We fully agree with the reviewer about the importance of reproducibility. Therefore, we have uploaded our data, the analyses code, and the results here: https://github.com/adam-sutton-1992/English-colour-terms-carry-gender-and-valence-biases-A-corpus-study-using-word-embeddings**

**We have also included a statement in an appropriate place of the method section (page 8 of the revised manuscript).**

R.1.3. I am somewhat familiar with NLP, word2vec etc., but I found it still hard to follow the way the results are presented. For one, the figures of the histograms in the supplementary materials should perhaps be part of the body of the paper. They are quite integral to understanding what’s going on, especially since much of the methods section talks about using 2SD and 3SD cut-off values from the distribution — without the actual distribution ever being shown in the paper!

**Reply: We agree with the reviewer that the supplemental figures improve the understanding of the method. Therefore, we have moved these figures to the main text, now Figures 1 and 2.**

R.1.4. Overall, I think that a lot of the presented numbers need to be “grounded”, that is, the reader wants to get a sense for the numbers involved, and what are large or small quantities. Given that the main topics of the paper (gender, color, valence) are of high relevance to psychologists, this audience will definitely want to see some measure of effect size that can readily be interpreted. Throughout the paper I was left wondering whether these were strong or weak biases.

**Reply: We understand what Dr Winter wishes us to add to the manuscript. However, we are dealing with data that cannot be handled or presented this way. Basically, we cannot calculate effect sizes, since we established biases in a corpus of text providing us with one data point per bias and per colour term. We have no participants, and do not know who created the texts, an individual or several individuals. That said, we believe that we can “ground” our scores, because we compare our scores with the scores of the 100,000 most frequent words.**

**To explain this reasoning still more concretely, in the results section, we report which colour terms had a larger bias than 95% of the most common words. These terms, we suggest, have an extreme bias. For instance, we explain on pages 8-9 that pink had a lower gender bias than 95% of the most frequent words, suggesting it was biased towards femininity. Furthermore, we compared the scores of these colour terms with the scores of four anchor terms (nun, priest, happy, sad), which are defined as particularly biased on the gender and valence axis, respectively.**

R.1.5. The inferential statistics are based on resampling (as far as I understood), which is quite cool, and it seems to make sense for this dataset. But even though I’ve used resampling techniques myself quite a bit, I felt that the logic of the resampling techniques on p. 8-9 could be explained in more detail, especially since many psychology-oriented readers will not be familiar with this.

**Reply: Here, we are not sure if Dr. Winter is referring to the comparison of the colour terms with the 100,000 most frequent words, or the pairwise comparisons we have deleted. In case of the former, we did not use resampling. We compared the biases of our colour terms to a distribution of biases of 100,000 most frequent words.**

R.1.6. I think it would also be helpful to present tables of the gender and valence bias values. While the ranking of the color words is inferable from Fig. 1, I think people would like to see the actual values and the ranking in a simple table.

**Reply: We agree and present these values in Table 1.**

R.1.7. Now, I like the approach that is presented in the paper, and it makes a lot of sense for me to do this for gender, since there are no available gender norms for color words. However, there *are* valence norms for color words, such as the widely used Warriner et al. (2013) dataset. I recognize that these are very different quantities from the ones presented in the text, as this is not text-inferred norms but humanly rated valence. That said, I think it’s important to consider this data, since it is there and readily available. I did a simple check and found that in fact all color words were biased towards positivity in this dataset. Pink, orange, and blue were among the most positive, and black is negative. A lot of this (although not all) mirrors the results obtained in this paper. I think correlating the values from the paper with this widely used dataset would be useful, if only for triangulating the results. Any differences that emerge could be theoretically interesting.

I attached the code and dataset used to reproduce this table in this zip file:

http://appliedstatisticsforlinguists.org/color_valence_papers_code.zip

**Reply: Our understanding is that Dr Winter suggests that we implement additional statistical analyses, which would compare our results with those of Warriner, Kuperman, and Brysbaert (2013). These authors provided “norms” of 13,915 English lemmas (words), including colour terms, based on participant ratings. When considering running and showing additional numerical comparisons, we asked ourselves why we would not also compare the current results with yet other existing datasets, including our recently published results from the International Colour-Emotion Association Survey (Jonauskaite et al., 2020). To make the long story (of our group reasoning) short, we decided to compare our current results with previous studies, including Warriner’s et al.’s and Saif’s (see comment R.1.8.), in the discussion and keep the comparisons descriptive. Otherwise, we would risk going too far from the original goal of the study to test valence and gender of *pink*, *red*, and *blue* in a corpus.**

**As an informal add-on to Dr Winter, his comments are relevant to our reasoning on our data more widely. We are likely to return to the data sets he suggests, hopefully in the near future. We started to look at the context of data collection (lab studies, large data sets), being also interested in whether results can be separated according to whether or not only native speakers added to the data. Firstly, we find negative valence for some colour terms in the lab, but mainly positive valence for datasets such as the one we used here. We feel it worthwhile understanding how different methods lead to different affective loadings. Yet, to do so, it is worthy focussing on all basic colour terms. Secondly, we are planning to look at our data or published data separating people who gave their answers in a survey / study performed in their native language or not. When we had a first look at an English data set in 2015, completed by native and non-native speakers, expected affective loadings were stronger for native speakers.**

R.1.8. There’s also some NLP work on word-color associations by Muhammad Saif that I think is worth incorporating, and he has a whole database of words that are associated with color terms. Perhaps one could do the reverse here and look for the gendered terms in this database and see whether they are associated with the colors.

https://www.aclweb.org/anthology/W11-0611.pdf

I’m not saying that the latter work has to be incorporated, but it’s worth acknowledging it. Incorporating the Warriner et al. (2013) data however is going to be a piece of cake, so perhaps worth doing.

**Reply: We would like to refer to our reasoning to the previous point by this reviewer (R.1.7). We also acknowledge that Dr. Winter’s idea is well appreciated, namely the question of bidirectionality. We have data to explore this question for colour-emotion associations, the perspective which also motivated the current study on the gender and affective valence of pink, blue and red.**

R.1.9. Theoretical framework

The paper cites a good deal of relevant research. I think it is missing out however on a bit of a stronger connection to cognitive science. Specifically, there are theoretical approaches within cognitive science that *predict* that biases found in psychological studies should be reflected in linguistic data, and work in cognitive science which looks at how language statistics in general reflect psychological biases and real-world patterns. Chiefly, Max Louwerse’s Symbol Interdependency Theory comes to mind. We review some of the relevant literature in section (iii) “Language statistics as knowledge” of this paper (pp. 4-5): https://royalsocietypublishing.org/doi/pdf/10.1098/rstb.2017.0137

I’m not saying that you have to cite our paper, but perhaps you find some of the references therein useful. I would think that by incorporating insights from this general view of language (as statistically mirroring psychological biases) into your work, you could show scope and increase the relevance of this paper to a wider audience.

**Reply: We agree that such and similar work is relevant to understand colour meaning. We have added these references to the introduction, on page 4** *“We wanted to learn whether above empirical results on gendered colours can, indeed, be observed when looking at gender and valence biases in widely available written texts. This should be the case according to theories in cognitive sciences [37,38].”*

**Furthermore, we just got an article accepted, in which we report on colour-emotion associations in a population of red-green colour-blind individuals (Jonauskaite et al., 2021, *PeerJ*). We found high consistency in such associations between colour-blind and non-colour-blind individuals, irrespective of whether they associated colour terms or colour patches with emotions. These findings highlight that conceptual associations are sufficient for such associations to occur, questioning the immediate role of direct perceptual experiences. We are planning to extend our work to blind individuals in the future.**

R.1.10. There’s also this paper that I think will turn out relevant to you:

Shepard, R. N., & Cooper, L. A. (1992). Representation of colors in the blind, color-blind, and normally sighted. Psychological Science, 3(2), 97-104.

It shows that semantic associations between colors are similar in the blind, even if they have never seen colors. This can only be explained by making recourse to language statistics.

**Reply: Absolutely, and please see our response to your previous point R.1.9. We see a strong enthusiasm for colour processing from a language perspective. While we appreciate all these thoughts and ideas, we wish to highlight that our interest comes from colour-emotion associations, and less so from colour naming and conceptual colour representations. All our work is linked to colour-emotion associations. One of the reasons, why we also felt we need to remind readers that the study focusses on the three gendered colours – pink, blue and red.**

R.1.11. Finally, I have attached two papers from our lab into the zip file above ( http://appliedstatisticsforlinguists.org/color_valence_papers_code.zip ), in one we look at gendered terms in natural language corpora and show that the frequency with which terms are mentioned as male/female significantly correlates with psychological biases from a gender bias rating study. The paper is at proof stage, but it is citeable — I think that this result is directly relevant to what you are doing.

**Reply: We thank Dr. Winter for sending this very interesting manuscript. We looked for a place to implement this study, first in the introduction, then in the discussion, but failed to see an appropriate place. Maybe, we missed an obvious link, Dr. Winter has in mind. Happy to adjust in case he wishes us to implement the reference at a particular place.**

R.1.12. When it comes to other cognitive science work, the finding that white > black in terms of positivity is interesting to me, and there’s much work this relates to. In the zip file, I attached a paper of mine that reviews the psychological work on black/white darkness related metaphors. And when you state that “black” is associated with sadness and death, it is critically also associated with fear (as evidenced by many studies).

**Reply: We are aware of the metaphorical meanings of black and white. While interesting and relevant to our overall line of work, we decided to keep our discussion focused on the three main colour terms of interest (*blue, pink, red*). During the revision, it became clear that we need to make it more obvious that our study assumptions originated from our former publication on gendered colour (Jonauskaite et al., 2019), and that we should make this more obvious. Thus, we lessened the importance of the other colours including *black* and *white*, and their metaphorical meanings. Even though we did not expand this section, we included the mention that *black* is also associated with *fear* on page 14 of the revised manuscript.**

R.1.13. Sorry for recommending that many of my own papers — there’s no need to cite them (the paper is good as is), but the references therein will surely prove useful for you to consider enlarging the scope of your work and incorporating some of the cognitive science work into your introduction and discussion. Your paper is quite on the short side of things anyway!

**Reply: We really appreciate the reviewer’s comments and welcome all suggestions that helps us enlarge the scope of our manuscript as well as stimulate thoughts for future studies. We have also extended the discussion.**

R.1.14. Other concerns

I agree that for large-scale corpora such as Wikipedia and newspapers, author and register variation etc. does not matter that much (as discussed in your limitations section). However, when it comes to the news in particular, it is quite likely that some of the metaphorical meanings of these words are quite dominant. For example, I can imagine that “black” in particular comes up in quite heated racial discussions, and some of its negativity associated with this word is either due to structural biases against blacks (e.g., reporting of crimes etc.) or newspaper articles discussing the negative topic of racism. Likewise, “green” in newspaper texts will almost always be associated with environmentalism. It’s perhaps worth noting more strongly that newspapers are by no means a ‘neutral’ sort of text type. You mention this in your limitations section a bit, but I think this could be emphasized more strongly, perhaps also with specific examples.

**Reply: We can relate to the examples by Dr. Winter, and incorporated them in the limitation section (pages 15-16) of the revised manuscript:**

*“Worth noting, neither our corpus study nor empirical studies reflect spontaneous conversations. When setting up research studies, researchers decide beforehand what they wish to test, designing the method accordingly. In the current and other corpus studies, one extracts meaning from large corpora. In our case, we used newswire and Wikipedia articles, conveying information that was likely filtered by topic, and presented in a socially and politically acceptable manner. If we take the terms black and green, the former might dominate discussions around racial and the latter around environmental issues. Furthermore, obvious descriptors might not be mentioned. A writer might not see the necessity to state that grass is green, or faeces are brown. Finally, the contents of corpora might depend on when and where they were published, as topics vary in popularity over time and between places. Most recently, gender issues were again intensively discussed with the prominence of the #MeToo movement.”*

R.1.15. Speaking of examples: a linguist reader would perhaps want to see SOME linguistic examples from the corpora to see how the gender/valence dynamics of these words pan out in actual discourse. This would make things MUCH more concrete for some readers.

**Reply: We considered how to extract and integrate linguistic examples of gender and valence dynamics of colour terms. However, we decided that we cannot provide readers with examples without speculating, because it is technically impossible (or very difficult) to know how the embeddings were calculated, considering we have 6 billion word-tokens. In analogy, we can think about machine learning methods. Some of these methods can predict a quality of interest but it is nearly impossible to know which predictors contributed the most or the least to the overall prediction.**

R.1.16. The paper is generally pretty good in terms of political correctness (important for this topic!), but I feel that there are two minor areas for improvement:

Include a statement somewhere that you recognize that gender is a non-binary concept. I’m all for using the categories ‘masculine’ and ‘feminine’ as this is arguably the primary division within conceptual gender space, but people from the LGTBQ+ community may find it offensive if the non-binariness of gender is not at least acknowledged (in the paper attached in the zip, we do this at length in footnote 1).

**Reply: We have added this acknowledgment in the footnote of the revised manuscript on page 6: “***We acknowledge that gender is non-binary. However, we treated gender for this data set as such, because we relied on pre-existing data, which separates gender in a binary fashion (i.e., “heshe” list of LIWC).”*

R.1.17. Some statements have to be hedged more to make it clear that you do not endorse them. This starts with the opening sentence “Many people would agree that *blue* is for boys and *pink is for girls*” — sounds like we should agree with this! Reframing things in terms of “stereotypes” is, I think, better: “In Western societies, blue is stereotypically associated with boys and pink is stereotypically associated with girls”. Likewise, “many parents are used to choosing pink when dressing their daughters” could be misread to sound as if this is a good habit. Just saying “many parents chose pink...” is better. Finally, “Pink further represents groups of low social power, such as children and homosexuals”, I would change to “... is stereotypically associated with groups ...”. Also, consider using another term for ‘homosexuals’, which some consider to be quite negative (it is not the term the community uses to refer to itself).

**Reply: We agree with Dr Winter and have updated the manuscript accordingly (see page 3 of the revised manuscript).**

R.1.18. Minor points

R.1.18.1. Supplementary materials: “that blue is 0.08 more positive than red” — but what is the metric here? Is this small or large?

**Reply: This part has been excluded from the revised manuscript. While the readers can see these differences, we make no mentions towards them because we are no longer performing pairwise comparisons.**

R.1.18.2. P. 3, “Empirical studies have demonstrated” — empirical studies with whom? This is super relevant here since this bias is presumably not universal and the study populations matter here.

**Reply: On page 3 of the revised manuscript, we now write:** *“Empirical studies, focused on colour preferences and colour connotations, have demonstrated that pink is considered to be a feminine colour and blue a masculine colour [3,8,10,11].”*

R.1.18.3. P. 3, “because gender (girl) repeatedly co-occurs with the actual color (*pink*)” -> The italics are used for words elsewhere, but if this is the actual color, why is this italicized? Also, “gender” (which is an abstract concept) cannot “co-occur” with a color... this is a bit of a category error.

**Reply: On page 3 of the revised manuscript, we now write:** *“If we think about pink, its gendered connotations might emerge not only because pink repeatedly occurs in feminine contexts visually, but also because it co-occurs with other feminine words in languages (e.g., pink is a girly colour).”*

R.1.18.4. P. 4, I’m not all that happy about capitalizing artificial intelligence and natural language processing. Also, do we really need to talk about the approach in terms of AI and isn’t this a bit buzz-wordy? At this stage, I think we can just identify the precise approach right away. NLP and AI are super common after all.

**Reply: We agree with Dr. Winter that these concepts should not be capitalised. We have made the necessary changes throughout the revised version of the manuscript. When it comes to the question whether we should refer to our method as artificial intelligence or not, we believe the term is appropriate. Word embeddings are made for the purpose of being input into artificially intelligent systems and can themselves be seen as part of artificial intelligence pipelines. What we do here with word embeddings to help understand the corpora is not the intended use of word embeddings, they are designed to work in NLP and AI tasks. Therefore, we believe the terms artificial intelligence and natural language processing are in our manuscript as defined precisely by those disciplines.**

R.1.18.5. P. 4, Should “nurse” and “housekeeper” be in italics?

**Reply: Indeed, we have changed these words to appear in italics in the revised version of the manuscript**

R.1.18.6. P. 5, “pushing its valence bias towards neutrality” -> Wait, if it occurs in highly positive and highly negative contexts, it’s not neutral, right? Do you perhaps mean the average?

**Reply: Dr. Winter is correct, a valence bias around 0 does not necessarily mean neutrality. We have paraphrased this sentence in the revised version of the manuscript (page 5):** *“]. We expected red to be embedded in both positive and negative contexts, pushing its valence bias towards zero. For its gender bias, we assumed red to represent power, and informed by the literature on gender stereotypes, power would represent masculinity.”*

R.1.18.7. P. 5, “the literature on gender stereotype*s*” — s missing

**Reply: Now corrected.**

R.1.18.8. P. 5 — you mention first “typically 300” then the precise number of dimensions. The first is vague (I immediately asked myself: so how many dimensions were used here?), and it’s a bit confusing to have them both so close to each other.

**Reply: We have improved the description in the revised version of the manuscript (page 5):** *“Each word within a word embedding is represented by a vector w of high dimensionality d (d = 300 in our study).”*

R.1.18.9. P. 6, “We can test words using the following cosine similarity function” — How is this a “test”? That’s a bit of a weird word here?

**Reply: Following Dr. Winter’s suggestion, we have updated the sentence (page 7):** *“We scored all words using the following cosine similarity function”*

R.1.18.10. P. 7, Can you avoid using the word “antithetical” which many non-native speakers won’t know?

**Reply: We have replaced “antithetical” with “opposite” throughout.**

R.1.18.11. P. 7, “of the two dimensions *(gender and valence)*” — clearer to add this right away

**Reply: On page 5 of the revised manuscript, we now write:** *“We also included four anchor words – two for the valence extremes (happy, sad) and two for the gender extremes (priest, nun).”*

R.1.18.12. P. 8 — this page and the section “Data Analysis” needs the most reworking. First, I took issue with “Therefore, we were able to test if any of our words of interest were statistical outliers”, which does not follow from the distributions being normal. One can test for outliers for any distribution!! It’s not that obtaining a particular distribution is intrinsically connected to outlier-detection. Also, it has to be flagged that 2SD and 3SD are “heuristic cut-offs” -> These are really arbitrary standard deviation values that we have historically inherited. You are using this as a heuristic criterion, and that’s ok, but I’d like the word “heuristic” to be used to make it clear that this is a definitional matter, depending on where one sets the boundary. At first when you said “the 100,000 most common words” it wasn’t clear to me where these come from. And when you reference “the population mean”, I’m not sure you can say this unless you really have the full population (which you don’t, since even a corpus is a sample).

**Reply: We could see the problems our writing entailed. We have rewritten the data analysis section substantially. We invite Dr. Winter to read pages 5-8 of the revised manuscript. We feel that our procedure description has substantially improved.**

R.1.18.13. P. 12, “numerically, but not statistically” — drop the “but not statistically” bit, since this seems to equate inferential statistics with statistics... which I’m not OK with ;-)

**Reply: Sure, we see the point. We are now avoiding any mention to statistics in the revised manuscript and instead talk about comparisons with a normative population of 100,000 most frequent words, which is what we did.**

R.1.18.14. P. 12, “while the findings on *blue* went against the notion that *blue* signals masculinity” — not sure... first, “signals” may not be the right word here, since what is in question is just whether language statistics reflect this bias. Second, absence of evidence is not evidence of absence, and so I wouldn’t say that these results go *against* this notion.

**Reply: We paraphrased the sentence in questions as such (page 13 of the revised manuscript): “***In contrast, findings on blue did not confirm a masculinity bias in our corpus, unlike previous empirical studies would have suggested [1,3,10,11].”*

Good stuff! Looking forward to seeing this paper revised and published!

**Reply: We thank Dr. Winter for all the insightful and thought-provoking comments. We hope the revised version of this manuscript will meet his expectations.**

**Reviewer: 2**

This article presents an analysis of the gender and valence bias of color terms through a Glove embeddings model fitted against Wikipedia and news. The authors find results that are consistent with previous results from experiments.

The methodology is appropriate and the presentation is good. However, I have some comments regarding the interpretation of results and some methodological details:

R.2.1. Results are framed as "in natural language" in general, but are only based on a combination of Wikipedia and news. The authors fairly mention this in their limitations section, but to generalize results to natural language it would be necessary to replicate the results with other corpora and/or languages. As there are pre-trained embeddings for many corpora and LIWC present in a wide variety of languages, it would not be hard for the authors to add replications that support their generalization to natural language. In case this is not possible, the abstract and conclusions of the article can be further contextualized to point that results only apply to one English corpus, without generalizing to natural language.

**Reply: We agree with the reviewer and have taken care to specify that our results only apply to the English language. We have added this clarification in the title, in the abstract, and also discussed it as a limitation in the discussion, on page 15:**

“*However, our method has at least two limitations. First, in our case, the text sources were in English, likely written by native and non-native speakers. Therefore, our results should not be generalised to other languages and populations.”*

R.2.2. Positive and Negative Affect in LIWC are treated as ends of a single dimension of valence, while in LIWC they are conceived as two variables that can co-occur, not just in text but also at the individual level. Even if they are not completely orthogonal, mapping valence as the subspace between PA and NA in the embeddings space induces a situation in which the middle point is not well defined. In the definition of valence in the paper, a word that has the same cosine similarity to the centroid of PA words as to the centroid of NA words is neutral, but this does not have to be the case. If the authors really want to analyze emotional valence, there is a large number of affective norms lexica that would allow them to approximate a mapping from the embeddings space to the dimension of valence as defined in those lexica. If they wanted to study something more general in the lines of evaluative meanings, other lexica like the General Inquirer would be more suitable than LIWC. I recommend the authors to revise the assumption of their calculation of valence bias and revise whether zero in their scale really means a neutral bias.

**Reply: We extracted two important points from this relevant comment. First, combining positive and negative embedding spaces into a valence bias. Second, the meaning of 0.**

**In regard to the first point, we changed the manuscript, so that we first present the results for each concept separately (male, female, positive and negative emotions) and only then present combined results. We argue that the current representation does not mislead the readers (see Figures 1 and 2, and Table 1). We agree with the reviewer that some colour terms had extreme biases on both dimensions. We highlighted these observations in the results and discussion sections.**

**In regard to the second point, the reviewer is correct that the zero point in gender and valence biases does not indicate that these terms are neutral in terms of gender or valence. However, we believe we are not making this claim in the manuscript. While rewriting the method and results sections, we further clarified our methodology: first, we established similarities to the concepts of male, female, positive, and negative emotions of the colour terms. Here, zero means, indeed, no similarity. Second, we calculated gender and valence biases. In these cases, the zero point can mean zero for similarities to both opposing concepts, or an equal bias for similarities to both opposing concepts. We observed that some colour terms were strongly biased i) towards positive and negative emotions (*black*), and i) concepts of male and female (*green*, *blue*, *brown*, and *black*).**

**Thanks to this comment, we now discuss this issue and results in the discussion of the revised manuscript, on page 12:**

*“First, we checked for extreme similarities of our colour terms to the concepts of male and female. We defined extreme word similarities when a colour term had a higher similarity with a concept than 95% of the 100,000 most frequent words in the corpus (i.e., normative word population). These comparisons revealed that i) red and blue were closer to the concept of male and ii) blue and pink were closer to the concept of female than 95% of words in the normative word population. That meant that blue was close to both concepts, yielding overall no gender bias. When looking at gender biases in comparison to the normative word population, pink was the only colour term with a gender bias. We confirmed its bias towards femininity. Against our predictions, neither blue nor red were biased towards masculinity and no other colour term had a strong gender bias. Hence, we concluded that only pink conveys gender biased information (femininity) in these texts.”*

R.2.3. The null model used for statistical analysis assumes that gender and valence bias are orthogonal. From previous works in sentiment analysis, we know that this is unlikely to be the case in many models like Glove. This assumed orthogonality can be seen in the Figure, where the limits for significance describe a square. Before concluding what associations are significant and which are not, the authors can use the bivariate distribution of valence and gender bias in their all-pairs sample and test each 2-D point of colors against that more realistic null model. This might very well add power to the method.

**Reply: We have deleted this figure from the revised version of the manuscript (also see our response to R.2.4.).**

R.2.4. The statistical test assumes normality of the distribution under the null, but the SI figure seems to show quite some deviation from a normal distribution, especially for the case of valence bias (if the lines in the figure are indeed a normal fit, which is not explained). I think the authors do not need this normal assumption at all, they can just use the quantiles of the bivariate distribution composed of the joint values shown in both SI figures, labelling for example which points are beyond the 95% quantiles in their color word analysis.

**Reply:  We completely agree with this point. Thus, we replaced our previous analyses, based on standard deviations, with the new analyses based on 95% quantiles. This approach did not change our results much.**

R.2.5. On a clarity note, authors should report which version of LIWC they used. They cite the 2001 version, which would be quite outdated compared to the 2015 version. They should also explain how they mapped entries in the LIWC dictionary, especially those with wildcards or other rules, to individual words in the embeddings model.

**Reply: The reviewer is right, we cited the LIWC 2001 version, while we had actually extracted word embeddings from the LIWC 2015 version. In the first version of the manuscript, we decided to cite the original LIWC work, as common in the field of artificial intelligence. Following the reviewer’s comment, we also cite the LIWC 2015 version.**

**Page 6 of the revised manuscript now reads:** *“We used the LIWC 2015 version [48]”*
